# Peach allergy: different clinical profiles across Europe

**DOI:** 10.1186/2045-7022-1-S1-S58

**Published:** 2011-08-12

**Authors:** Montserrat Fernandez-Rivas

**Affiliations:** 1 Hospital Clínico San Carlos, Servicio de Alergia, Madrid, Spain

## 

Rosaceae fruit allergy is the most common food allergy in Europe in patients over 5 years of age. The two fruits most frequently involved are peach and apple. Different clinical phenotypes of peach allergy are observed across Europe in relation to different allergen sensitisation patterns to the peach allergens Pru p1 (Bet v 1 homologue), Pru p 3 (peach lipid transfer protein, LTP), and Pru p 4 (peach profilin). In areas rich in birch trees of Central and Northern Europe peach allergy is linked to birch pollinosis and apple allergy, and the major allergen involved is Pru p 1. These patients present mild oropharyngeal symptoms (oral allergy syndrome, OAS) upon peach ingestion. Profilin co-sensitisation is also found in a minority of patients, but the clinical presentation is similarly OAS. In contrast, in the Mediterranean areas free of birch trees peach allergy may result from a primary sensitisation to Pru p 3, or be linked to pollen allergy with sensitisation to pollen profilin and secondary to Pru p 4, or a combination of both. In patients monosensitised to Pru p 3 the phenotype is severe and the great majority experience systemic reactions including anaphylaxis. In subjects monosensitised to Pru p 4, peach allergy is linked to a pollen allergy, generally to grass pollen, and the phenotype is mild with OAS in all the patients. When sensitisation to Pru p 3 and Pru p 4 are combined systemic reactions are less frequently observed than in patients monosensitised to Pru p3, and OAS is more frequently reported. Sensitisation to Pru p 2, the thaumatin like protein in peach, may be found in combination with Pru p 3 and 4, but its clinical relevance has not yet been established. Peach allergy across Europe is an interesting model food allergy that illustrates how different allergen sensitisation profiles determine the clinical phenotypes of fruit allergy. The reason for the (almost) lack of sensitisation to Pru p 3 in Central and Northern Europe still remains unclear.

